# Factors Controlling the Stable Nitrogen Isotopic Composition (δ^15^N) of Lipids in Marine Animals

**DOI:** 10.1371/journal.pone.0146321

**Published:** 2016-01-05

**Authors:** Elisabeth Svensson, Stefan Schouten, Ellen C. Hopmans, Jack J. Middelburg, Jaap S. Sinninghe Damsté

**Affiliations:** 1 Department of Marine Organic Biogeochemistry, NIOZ Royal Netherlands Institute for Sea Research, Den Burg (Texel), The Netherlands; 2 Department of Earth Sciences, Faculty of Geosciences, Utrecht University, Utrecht, The Netherlands; University of Otago, NEW ZEALAND

## Abstract

Lipid extraction of biomass prior to stable isotope analysis is known to cause variable changes in the stable nitrogen isotopic composition (δ^15^N) of residual biomass. However, the underlying factors causing these changes are not yet clear. Here we address this issue by comparing the δ^15^N of bulk and residual biomass of several marine animal tissues (fish, crab, cockle, oyster, and polychaete), as well as the δ^15^N of the extracted lipids. As observed previously, lipid extraction led to a variable offset in δ^15^N of biomass (differences ranging from -2.3 to +1.8 ‰). Importantly, the total lipid extract (TLE) was highly depleted in ^15^N compared to bulk biomass, and also highly variable (differences ranging from -14 to +0.7 ‰). The TLE consisted mainly of phosphatidylcholines, a group of lipids with one nitrogen atom in the headgroup. To elucidate the cause for the ^15^N-depletion in the TLE, the δ^15^N of amino acids was determined, including serine because it is one of the main sources of nitrogen to N-containing lipids. Serine δ^15^N values differed by -7 to +2 ‰ from bulk biomass δ^15^N, and correlated well with the ^15^N depletion in TLEs. On average, serine was less depleted (-3‰) than the TLE (-7 ‰), possibly due to fractionation during biosynthesis of N-containing headgroups, or that other nitrogen-containing compounds, such as urea and choline, or recycled nitrogen contribute to the nitrogen isotopic composition of the TLE. The depletion in ^15^N of the TLE relative to biomass increased with the trophic level of the organisms.

## Introduction

Stable isotopes of carbon and nitrogen (δ^13^C and δ^15^N) are routinely used in ecology to study a wide range of subjects such as trophic interactions, energy flow, diet composition, feeding habits, and migration (see e.g. [1,2]; and references therein). Stable carbon isotopes are generally used to distinguish between energy sources, such as terrestrial vs. aquatic, because differences in δ^13^C values are generated during primary production and largely conserved during heterotrophic processing (e.g. [3,4]). Nitrogen isotopes are mainly used to infer trophic transfers as each trophic step results in an increase in the δ^15^N signal of biomass (e.g. [5,6]). More recently, the δ^15^N of specific amino acids has been used to estimate trophic levels [7–9].

For application of stable isotopes in trophic ecology, lipids are sometimes removed from bulk biomass prior to stable isotope analysis. This is done because lipids are depleted in ^13^C compared to proteins and carbohydrates due to fractionation during lipid biosynthesis and because different tissues and organisms have variable lipid contents [10,11]. Interestingly, it has frequently been observed that lipid extraction also result in changes in the δ^15^N of the residual biomass [12–15]. The change in δ^15^N bulk biomass with lipid extraction (Δδ^15^N_residue-bulk_) can vary substantially (-2 to + 2.1 ‰ [1]), but unlike the change in δ^13^C from lipid extraction (e.g. [16–18]), no clear relationship between Δδ^15^N_residue-bulk_ and parameters such as lipid content or C:N ratio of the organisms has been found. The relatively large range in Δδ^15^N_residue-bulk_ is problematic as the change caused by lipid extraction is similar in magnitude to reported trophic fractionation (e.g. [6,19]) and diet-tissue discrimination factors (e.g. [6,15,20]). There is uncertainty about the cause for these changes. Some studies hypothesized that co-extraction of lipid-bound proteins leads to the removal of some amino acids (e.g. [13]), although this would imply that the extracted amino acids had a strongly different nitrogen isotopic composition compared to the remaining amino acids [14]. Another hypothesis is that lipid extraction leads to removal of cellular waste products (e.g. ammonia), which have quite different nitrogen isotopic compositions than that of organic nitrogen [21]. However, no experimental evidence has been provided to support these hypotheses.

In this study we investigate the cause of the changes seen in the δ^15^N following lipid extraction of tissues of several marine animals by determining the δ^15^N of bulk and residual, lipid-free, biomass as well as of the total lipid extract (TLE). In addition, we identify the intact polar lipids present in the lipid extract to elucidate the sources of nitrogen to the lipid extracts. Finally, we determine the δ^15^N of amino acids to show that δ^15^N of the total lipid extract relates to the δ^15^N of serine and the source amino acid phenylalanine.

## Materials and Methods

### 2.1 Samples

Four species of benthic invertebrates (Common cockle, *Cerastoderma edule*; Pacific oyster, *Crassostrea gigas*; Green shore crab, *Carcinus maenas*; and lugworm, *Arenicola marina*) and three fish species (Atlantic herring, *Clupea harengus*; Brown trout, *Salmo trutta*; and Twait shad, *Alosa fallax*) were collected in the Dutch Wadden Sea in 2011. Fishes were collected using a passive fishing device (kom-fyke net; [22]) in the Marsdiep area near the southern part of Texel (Netherlands), and at the entrance to the western Dutch Wadden Sea, in spring (April to June) and autumn (September to October) of 2011. Individuals were either processed or frozen immediately after capture. All fish were measured to the nearest cm total length and weighed, and gill tissue and white muscle were sampled (see [18]). Muscle tissue was taken from below the dorsal fin and cleaned from skin and scales. Invertebrates were collected through the SIBES (Synoptic Intertidal Benthic Survey) program (https://www.nioz.nl/sibes). Animals from sediment cores were collected, cleaned, and identified. Depending on the type and size of the animal, different types of tissue samples were collected from each individual (e.g. muscle, head, or whole), which were then frozen, followed by freeze-drying for 72 h in glass vials, and stored at -20°C until further processing. Before lipid extraction and stable isotope analysis, samples were homogenized using a mortar and pestle or a ball mill grinder (Retsch, Düsseldorf, Germany).

### 2.2 Lipid extraction (TLE)

Total lipid extracts (TLE) were prepared as described in Svensson et al. [18]. In short, samples were extracted four times using dichloromethane (DCM) and methanol (MeOH) (2:1 v/v) and ultrasonication (1x10 min plus 3x5 min) and centrifuged at 1000xg, 2.5 min. Organic solvents were pipetted off after each extraction and combined as the total lipid extract (TLE). Residual biomass (lipid free) and TLEs were evaporated to dryness under a gentle stream of N_2_ at room temperature.

### 2.3 Intact polar lipid analysis

Intact polar lipids (IPLs) were analyzed using HPLC/ESI/MS according to Sturt et al. [23] with some modifications as described in Schouten et al. [24] and Bale et al. [25]. In short, lipid extracts were re-dissolved in hexane:2-propanol:water (72:27:1, v/v/v) at a concentration of 2 mg mL^-1^ and filtered through a 0.45 μm regenerated cellulose (RC) filter (Alltech Associates Inc., Deerfield, IL) prior to injection. An Agilent 1200 series LC (Agilent, San Jose, CA), equipped with thermostatted auto-injector and column oven, and coupled to a Thermo LTQ XL linear ion trap with Ion Max source with electrospray ionization (ESI) probe (Thermo Scientific, Waltham, MA), was used. Separation was achieved on a LiChrospher diol column (250 x 2.1 μm, 5 μm particles; Alltech Associates Inc., Deerfield, IL) maintained at 30°C. The following elution program was used with a flow rate of 0.2 mL min^-1^: 100% A for 1 min, followed by a linear gradient to 66% A: 34% B in 17 min, maintained for 12 min, followed by a linear gradient to 35% A: 65% B in 15 min, where A = hexane:2-propanol:formic acid:NH_3aq_ (14.8M) (79:20:0.12:0.04, v/v/v/v) and B = 2-propanol:water:formic acid:NH_3aq_ (14.8M) (88:10:0.12:0.04, v/v/v/v). Total run time was 60 min with a re-equilibration period of 20 min in between runs. The lipid extracts were analyzed by an MS routine where a positive ion scan (*m/z* 400–2000) was followed by a data dependent MS^2^ experiment where the base peak of the mass spectrum was fragmented (normalized collision energy 25, isolation width 5.0, activation Q 0.175). This was followed by a data dependent MS^3^ experiment where the base peak of the MS^2^ spectrum was fragmented under identical fragmentation conditions. This process was repeated on the 2^nd^ to 4^th^ most abundant ions of the initial mass spectrum. Major IPL classes were identified as described in Brandsma et al. [26].

### 2.4 Stable isotope analysis

The stable nitrogen isotopic ratio was determined on bulk biomass (δ^15^N_bulk_) and on residual biomass after extraction (δ^15^N_residue_), as well as on the total lipid extracts (δ^15^N_TLE_). For δ^15^N_bulk_ and δ^15^N_residue_, ca. 0.4–0.8 mg of freeze dried, homogenized sample was weighed into tin cups. These samples were analyzed for δ^15^N and percent total organic carbon (%TOC) and percent total nitrogen (%TN) content in duplicate using isotope ratio monitoring mass spectrometry (IRMS) with a Delta V Advantage-IRMS coupled to a Flash 2000 elemental analyzer (Thermo Scientific). Total lipid extracts were dissolved in ethyl acetate and pipetted into tin cups for a final weight of ca. 0.4 mg for δ^15^N determination. Ethyl acetate was allowed to evaporate completely at room temperature (minimum 6 h) before folding the cups for analysis. Due to the low amount of nitrogen compared to carbon in the lipid extracts, the TLE fraction was analyzed for δ^15^N on a Delta XL isotope ratio MS (Thermo Finnigan) coupled to a Flash 1112 Series elemental analyzer (CE Instruments) equipped with a liquid nitrogen trap to remove CO_2_ from the sample stream.

Stable isotope ratios are expressed using the δ notation in units per mil according to:

$$\delta \left(‰\right)\text{=}\frac{{\text{R}}_{\text{sample}}}{{\text{R}}_{\text{standard}}}\text{-1}$$

where R = ^15^N/^14^N, and expressed in per mil versus air. An acetanilide standard with a δ^15^N value of 1.18‰ (standard obtained from Arndt Schimmelmann, Indiana University; [27]), and known %TOC and %TN content, was used for calibration. The average repeatability of δ^15^N determination was 0.2‰ based on repeated analysis of the acetanilide standard. The pooled standard deviation of replicate measurements (n = 2–5) were ≤0.6 ‰ for bulk and lipid-free biomass, and ≤0.9‰ for total lipid extract.

### 2.5 Amino acid nitrogen isotope analysis

The method used for amino acid isotope analysis was a modified version of the method from Metges et al.[28] and Chikaraishi et al. [29]. In short, tissue samples were hydrolyzed in 6M HCl at 110°C for ca. 18 h. Hydrolyzates were then filtered (GHP Nanosep centrifugal filtration devices, Pall Co), and defatted using n-hexane:DCM (3:2, v/v). Samples were dried under a flow of N_2_ with repeated additions of methanol. Carboxylic acid groups of amino acids were esterified by addition of 200 μl 2-propanol:acetyl chloride (4:1, v/v) and heating at 110°C (2 h). Amine groups were subsequently acetylated using DCM:pivaloyl chloride (200 μl; 4:1,v/v) at 110°C (2 h). After each derivatization step, any leftover reagents were removed by addition of DCM and drying under a gentle stream of N_2_ (2x). Derivatized amino acids were dissolved in ca. 200 μl bidistilled water and extracted using n-hexane:DCM (3:2, v/v), dried over MgSO_4_, diluted to a suitable concentration with dried (MgSO_4_) and de-gassed DCM, and stored at -20°C in amber vials until analysis.

The N-pivaloyl/2-propyl derivatives of amino acids were analyzed by gas chromatography/combustion/isotope ratio mass spectrometry (GC/C/IRMS) using a Thermo Delta V Advantage connected to an Agilent 6890 GC. The GC and IRMS were interfaced via a combustion furnace (980°C), reduction furnace (650°C), and a liquid nitrogen cold trap to remove CO_2_. Separation of the derivatized amino acids was achieved on a DB-5ms column (Agilent J&W, 60 m x 0.32 mm i.d., 0.50 μm film thickness; Agilent Technologies), using the following temperature program: Initial temperature 70°C for 1 min; ramp up to 140°C at 10°C min^-1^, dwell for 5 min; ramp up to 190°C at 2°C min^-1^; ramp up to 300°C at 10°C min^-1^, hold for 10 min. Carrier gas was helium at a continuous flow of 2 ml min^-1^ (29 cm s^-1^). Injection volumes ranged from 0.4–2 μl. An in-house standard mixture consisting of five amino acids (glycine, norleucine, glutamic acid, phenylalanine, and tyrosine) with known δ^15^N values (determined offline) was used to evaluate daily system performance. Long-term reproducibility, based on the standard deviation of multiple injections (n = 52) of the in-house standard mixture was 0.7 (glycine), 0.9 (norleucine), and 1.2‰ (phenylalanine, glutamic acid, and tyrosine).

### 2.6 Data analysis

Correlations were evaluated using Pearson correlation analysis. The non-parametric Kruskal-Wallis test was used to evaluate differences in isotopic compositions, because of the relative small dataset. For the few cases with larger sample size, a Student t-test was used and the results were similar. Data were non-transformed and evaluated at a 5% significance level. Statistical tests were done using XL-Stat version 2015.4.01.20780.

Data used in this study are also available at http://doi.pangaea.de/10.1594/PANGAEA.855456

### 2.7 Ethical statement

This study was done with permission from the Dutch Fisheries Inspection of the Ministry of Agriculture, Nature and Food Quality (Visserijinspectie) and reviewed and approved by the Animal Experiments Committee (Dierenexperimentencommisie, DEC) under DEC protocol NIOZ 2010.03.

## Results and Discussion

### 3.1 δ^15^N contents of extracted biomass and lipid extracts

**Fig 1:**
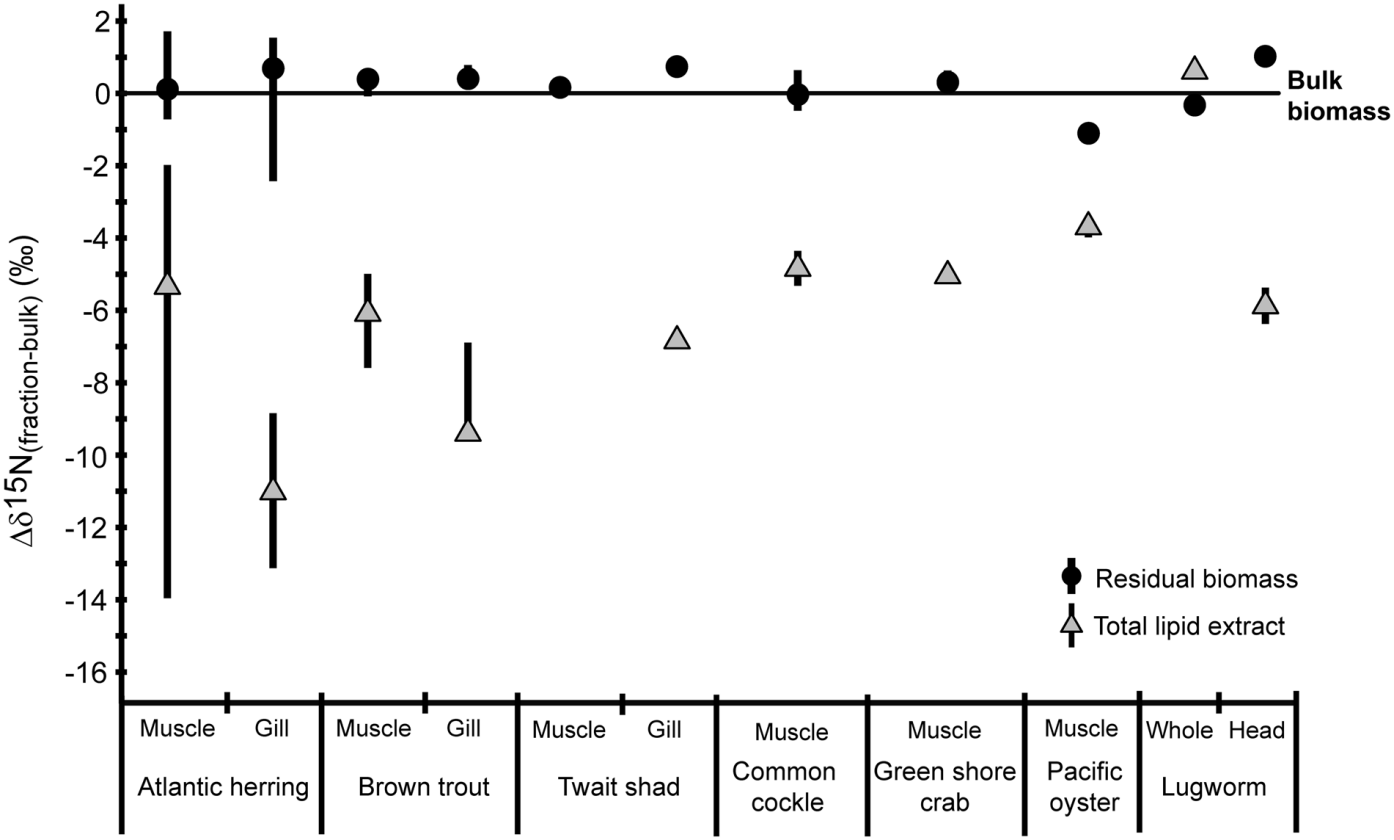
δ^15^N values of residual biomass, the total lipid extract and serine normalized to bulk biomass. Median values (symbols) and ranges of differences in δ^15^N of residual biomass, the total lipid extract (TLE) and serine compared to bulk biomass (Δδ^15^N_fraction-bulk_) for the different animals.

Lipids were extracted from biomass of several species of aquatic animals and the δ^15^N values were determined both before (bulk biomass) and after lipid extraction (residual, lipid-free biomass), as well as that of the total lipid extract (TLE). Bulk biomass δ^15^N values for different species and tissue types ranged, on average, from 6.7 to 17‰ (Table 1). The δ^15^N of residual biomass after lipid extraction differed by -0.9 to + 1.8 ‰ compared to bulk biomass (Δδ^15^N_residue-bulk_; Fig 1 and S1 Table) which is consistent with previous studies [30,31].

**Table 1:** Ranges of δ^15^N values and C:N ratios for bulk biomass, residual (lipid-free) biomass and lipids (total lipid extract) per species and tissue type. n = number of individuals analyzed. Standard deviation of δ^15^N values from replicate measurements were for bulk and lipid-free biomass ≤0.6 ‰, for total lipid extract ≤0.9.

		Bulk biomass				Residual biomass			Total lipid extract		
Species	Tissue	n	%lipids	δ^15^N	C:N	n	δ^15^N	C:N	n	δ^15^N	C:N
Atlantic herring	Gill	6	19–32	10.6–14.4^a^	4.8–8.0^a^	6	10.7–13.7	3.0–3.3	4	0.4–9.2	62.9
	Muscle	6	7–40	11.4–16.2^a^	3.3–6.1^a^	6	11.4–16.3	2.4–3.1	5	-0.5–3.1	14.6–23.2
Brown trout	Gill	6	4–11	13.9–16.3^a^	3.7–4.0^a^	6	14.2–16.7	3.2–3.5	3	7.2–11.0	19.5–42.2
	Muscle	7	5–25	13.7–16.5^a^	3.2–5.1^a^	7	14.4–17.0	3.0–3.2	5	4.3–9.7	21.3
Twait shad	Gill	2	6–9	14.7–16.7^a^	4.2–4.3^a^	2	14.8–17.0	3.3–3.6		n.d	n.d
	Muscle	2	6–7	15.8–17.1^a^	3.2–3.2^a^	2	16.7–17.9	3.1–3.1	1	10.3	14.1
Green shore crab	Muscle	3	2–6	13.2–15.8	3.7–5.3	3	13.4–16.5	3.3–4.4	1	8.2	n.d
Common cockle	Muscle	5	4–5	11.3–12.6	4.1–4.9	5	11.7–12.7	3.7–4.3	2	6.9–7.2	n.d
Pacific oyster	Muscle	2	2–6	12.3–12.3	3.0–3.3	2	13.3–13.5	3.2–3.3	2	6.0–7.0	n.d
Lugworm	Head	2	3–5	11.7–12.4	4.2–4.7	2	10.8–11.3	3.7–4.0	2	8.4–8.5	n.d
	Whole	1	1	6.7	3.1	1	6.6	3.5	1	7.4	n.d

^a^ Data from Svensson et al. [18].

n.d. = not determined.

The TLEs were depleted in ^15^N compared to bulk biomass in all samples except one (whole lugworm, Δδ^15^N_TLE-bulk_ = +0.7‰), with Δδ^15^N_TLE-bulk_ values ranging from -14 to +0.7‰ (Fig 1, Table 1, and S1 Table). The lipid content of the investigated tissues ranged from 1–40% with the majority having lipid contents ≤ 10% and lipids thus form a relative small portion of the total biomass. Furthermore, the relative amount of N in the lipid extract was small in most cases (average C:N ratio ranging from 14 to 63; Table 1) compared to bulk biomass (C:N ratio ranging from 3–8; Table 1) and highly variable. No correlation was observed between Δδ^15^N_residue-bulk_ and either the C:N ratio or %lipid of bulk biomass (Pearson correlation, r = -0.130 [P = 0.417], n = 40, and 0.239 [P = 0.132], n = 40, respectively).

Although there is a large variability in ^15^N of the lipid extracts, on average they were significantly depleted in ^15^N compared to biomass by approx. -7‰ (Kruskal-Wallis test, p<0.001, n = 26). The Δδ^15^N_residue-bulk_ of lipid-free biomass also varies, but the residue was on average significantly enriched in ^15^N by 0.4‰ (Kruskal-Wallis test, p<0.001, n = 41) compared to the original biomass. This suggests that, in general, lipid extracts contain ^15^N depleted nitrogen, which induces small but significant changes in ^15^N in the residual biomass. Remarkably, however, there is a large variability in both δ^15^N and C:N ratio of lipid extracts as well as Δδ^15^N_residue-bulk_ (Table 1). This may suggest that there is a mixture of different nitrogen sources in the lipid extract, such as lipids, lipid-bound protein and urea [12–14], all present in variable ratios and with different δ^15^N values. Below we investigate the δ^15^N of lipids as a potential source for the variable depletion in ^15^N in the lipid extract.

### 3.2 Sources of ^15^N depleted nitrogen in lipid extracts

**Fig 2:**
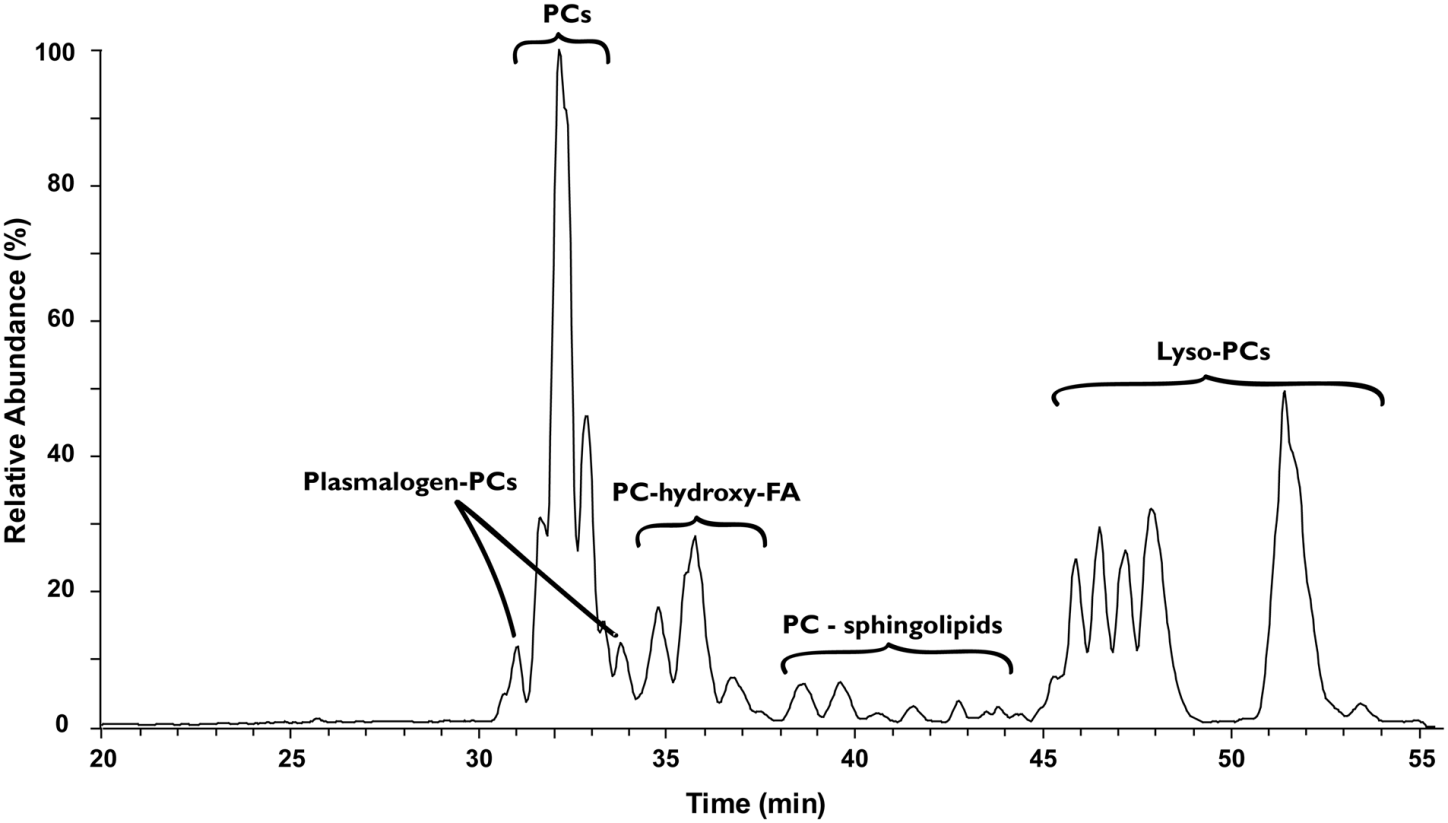
LC–MS chromatogram showing identified intact polar lipids in brown trout (*Salmo trutta*) gill tissue. Base peak LC–MS chromatogram (Gaussian smoothed) of MS^1^ of intact polar lipids (IPLs) in brown trout (*Salmo trutta*) gill tissue showing the prevalence of the nitrogen-containing IPL phosphatidylcholine. PC = phosphatidylcholine.

**Fig 3:**
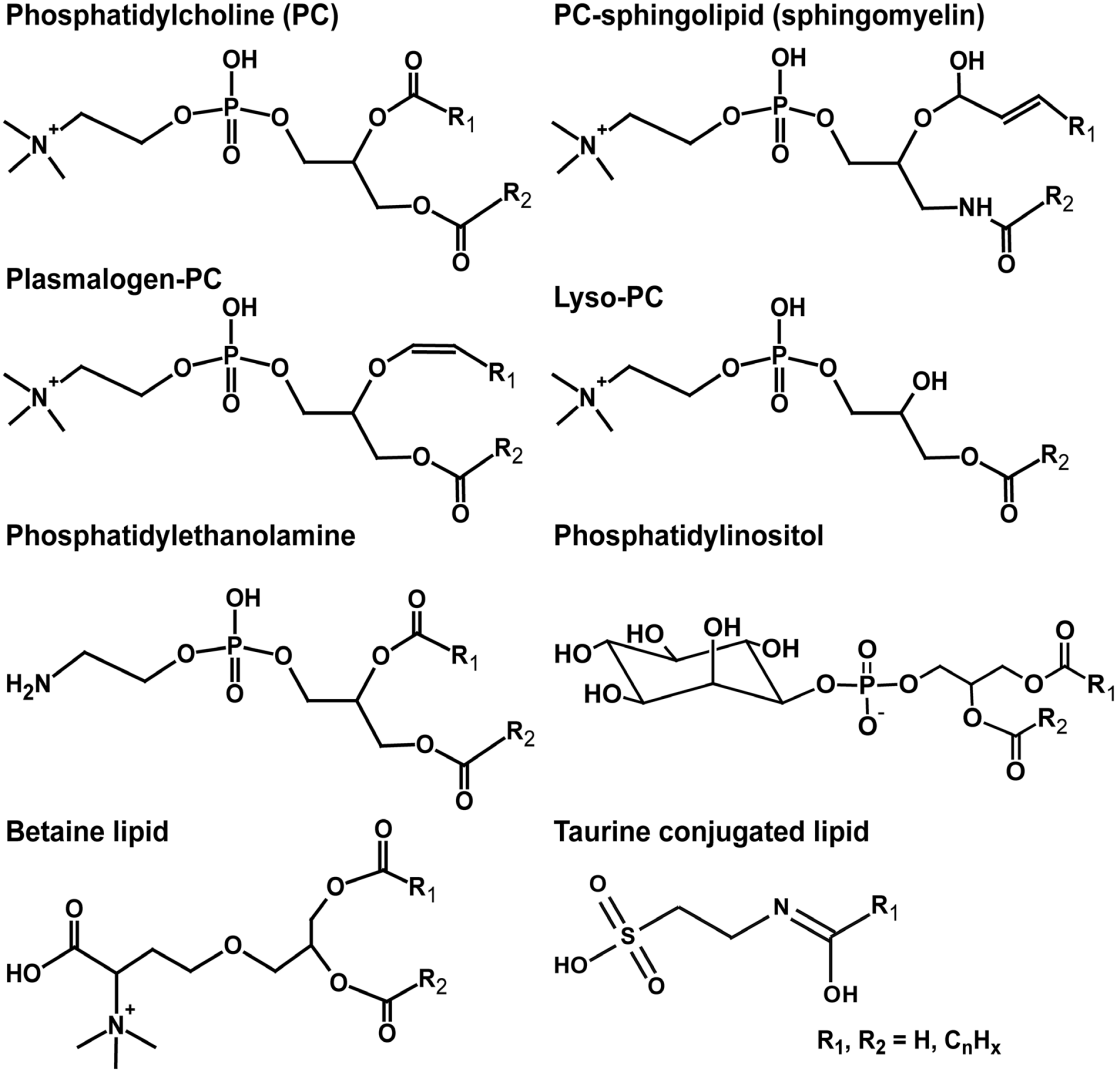
Structures of identified intact polar lipids in lipid extracts of animal tissues. PC = phosphocholine.

To investigate the origin of the ^15^N depleted nitrogen in the TLE we investigated its lipid composition by HPLC/ESI/MS. The majority of identified lipids in the TLEs comprised the nitrogen-containing phosphatidylcholines (PC; Fig 2 and S2 Table), which is a common lipid class in animal tissue (e.g. [32]). Taurine conjugated lipids as well as phosphatidylethanolamines (PE), betaines, and phosphatidylinositols (PI) were also detected in some species. With the exception of PI, all of these lipids contain one nitrogen atom in the lipid headgroup (Fig 3). The dominance of nitrogen-containing lipids suggests that they form an important source of nitrogen to the lipid extracts, although a contribution of nitrogen from other sources, such as extractable proteins and/or nitrogen containing waste products, cannot be excluded. Some evidence for the latter comes from the C:N ratio of the lipid extracts: the C:N ratio of some lipid extracts (Table 1) were lower than the theoretical C:N ratios of the identified lipids (25–50) suggesting the presence of additional nitrogen.

To assess whether the N-containing headgroups, in particular PC, were ^15^N-depleted compared to bulk biomass, as observed for the lipid extracts, the biosynthetic source of nitrogen for these headgroups was considered. Headgroups like PC and PE derive their nitrogen either from amino acids, choline or from recycled nitrogen within the cell [33,34]. The amino acid serine in particular is interesting as it is one of two main sources of cellular nitrogen to many nitrogen-containing lipids (the other being choline), while for PE it is the only nitrogen source. If serine contributes a large fraction of nitrogen to the lipids during biosynthesis, the δ^15^N values of the precursor serine should be reflected in the lipid product. We, therefore, determined the δ^15^N of serine in a selection of tissues (Table 2). Serine was indeed depleted in ^15^N compared to bulk biomass (Fig 1) with Δδ^15^N_ser-bulk_ ranging from -1 to -8‰. This agrees with previous observations in animals that serine generally is ^15^N depleted compared to total biomass (e.g. [35–37]). The range of depletion in ^15^N of serine was highly variable and resembled the variation in Δδ^15^N_TLE-bulk_ of the total lipid extract. Indeed, the Δδ^15^N_ser-bulk_ of serine is strongly and significantly correlated with Δδ^15^N_TLE-bulk_ of TLE (Pearson correlation, r = 0.94, [P<0.001], n = 8). The strong correlation between Δδ^15^N_ser-bulk_ and Δδ^15^N_TLE-bulk_ is in line with the biochemical evidence that serine is indeed an important source of nitrogen to the lipid pool and shows as well that the δ^15^N of lipid extracts were mainly determined by the δ^15^N of N-containing lipids. It should be noted, however, that the extent of depletion of serine was less than for the lipids by 3–4‰ in some tissues (Fig 1). These differences in δ^15^N between serine and lipid extracts may be due to one or a combination of several factors: (i) a (large) contribution of nitrogen recycled within the cell or originating from dietary choline, (ii) fractionation during biosynthesis of the headgroups from serine or choline. Further research using e.g. compound specific analysis of headgroups of lipids may shed further light on this.

**Table 2:** δ^15^N values of amino acids. Avg and s.d. = average and standard deviation of n injections. ALA = alanine; ASP = aspartic acid; GLU = glutamic acid; GLY = glycine; ILE = isoleucine; LEU = leucine; LYS = lysine; OH-PRO = hydroxy-proline; PHE = phenylalanine; PRO = proline; SER = serine; THR = threonine; TYR = tyrosine; VAL = valine.

	Brown trout muscle			Brown trout gill			Atlantic herring muscle			Atlantic herring gill			Green shore crab			Pacific oyster			Lugworm head			Lugworm whole		
	n	Avg	s.d.	n	Avg	s.d.	n	Avg	s.d.	n	Avg	s.d.	n	Avg	s.d.	N	Avg	s.d.	n	Avg	s.d.	n	Avg	s.d.
**ALA**	6	29.3	0.9	8	27.5	0.6	4	26.3	0.9	3	22.5	0.5	5	23.3	2.2	4	23.0	0.7	3	20.9	0.9	4	18.1	1.4
**ASX**	5	22.6	1.1	8	21.4	0.7	3	21.7	0.7	3	18.0	0.9	5	20.2	1.3	4	19.9	0.3	3	20.0	1.7	4	18.2	1.2
**GLU**	6	26.5	0.7	8	27.2	0.5	4	26.6	1.0	3	23.9	0.5	5	24.1	0.9	4	22.7	0.2	3	19.4	1.1	4	17.4	0.9
**GLY**	6	7.6	0.8	8	8.8	0.3	4	5.0	0.8	3	5.2	0.9	5	10.0	1.4	4	9.8	0.6	3	9.9	0.9	4	9.1	1.0
**ILE**	5	26.0	0.8	5	25.7	0.6	3	25.7	0.1	n.d.			5	18.3	1.8	4	19.1	0.7	3	16.7	0.7	2	16.2	1.4
**LEU**	6	26.3	0.7	8	26.9	0.8	4	25.8	0.7	3	21.9	1.1	5	19.8	1.9	4	19.1	0.8	3	17.7	0.5	5	16.7	0.8
**LYS**	6	4.0	0.8	8	2.8	0.3	4	5.0	0.4	n.d.			n.d.			4	8.0	0.8	3	3.6	0.5	2	2.1	0.2
**OH-PRO**	n.d.			8	22.9	0.9		n.d.		3	20.5	1.8	n.d.			n.d.			n.d.			n.d.		
**PHE**	6	8.6	0.9	6	9.9	0.8	3	7.6	1.1	3	6.0	2.4	5	8.9	1.0	4	11.7	0.7	3	9.3	0.8	2	11.0	0.5
**PRO**	n.d.			6	27.6	0.7	n.d.			n.d.			n.d.			n.d.			n.d.			n.d.		
**SER**	3	7.7	0.5	6	8.4	0.7	3	5.9	1.0	2	8.3	0.5	2	10.7	1.6	4	10.3	0.9	3	8.4	1.5	2	8.8	1.0
**THR**	5	-15.3	3.2	2	-22.2	0.0	3	-17.5	2.4		n.d.		1	-4.7		1	4.6		2	4.7	1.6	2	3.0	0.2
**TYR**	6	15.5	1.5	8	15.5	0.7	4	14.0	0.9	3	8.7	0.8	5	11.4	0.5	4	15.0	1.1	3	11.3	0.3	2	11.6	0.9
**VAL**	7	27.6	0.9	8	28.5	1.4	4	27.2	1.2	3	23.2	1.1	5	21.2	2.3	4	22.4	0.5	3	19.6	1.2	4	18.5	2.3

n.d. = not detected.

**Fig 4:**
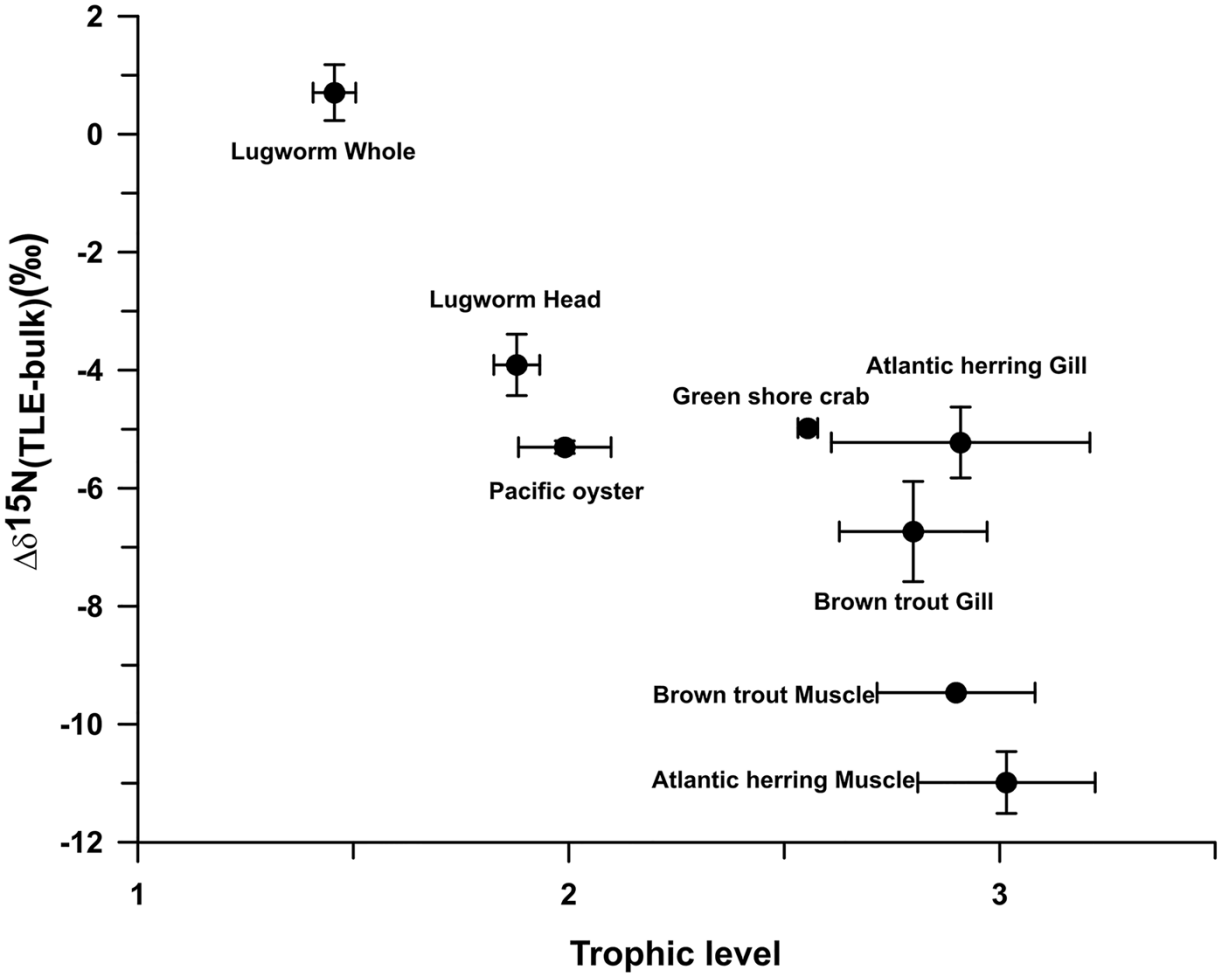
Difference in δ^15^N of lipid extracts (TLE) and bulk biomass (Δδ^15^N_TLE-bulk_) plotted against trophic levels. Δδ^15^N_TLE-bulk_ data points represent averages of several individuals with the error bar reflecting the standard deviation of multiple individuals (S1 Table). Trophic level data points are plotted as averages (with error) of replicate analysis of amino acids of a single individual. Trophic levels were calculated according to Chikaraishi et al [29] using δ^15^N values of the amino acids phenylalanine and glutamic acid.

The cause for the observed large variability in the ^15^N depletion of N-containing lipids is not clear. However, it is noticeable that the organisms with higher trophic levels, such as fish, show a larger depletion in ^15^N than those associated with lower trophic levels, such as lugworms. Indeed, when we calculate trophic levels for the different organisms based on the δ^15^N of glutamic acid and phenylalanine, using the equation of Chikaraishi et al. [29] for marine food webs, we observe an increasing depletion in ^15^N of the lipid extract relative to biomass with increasing trophic level (Fig 4). Further studies are needed to explore the cause for the large variability in the ^15^N depletion of N-containing lipids and whether the observed correlation with trophic level is causal.

## Conclusions

Our study shows that lipid extracts are generally highly depleted in ^15^N compared to bulk biomass. The majority of lipids in the lipid extracts from biomass in this study consisted of glycerophospholipids with a phosphatidylcholine headgroup, a nitrogen-containing lipid which is commonly found in animal tissues. The ^15^N depletion in the lipids is likely a reflection of the δ^15^N of the biosynthetic nitrogen sources such as the amino acid serine, which is also depleted in ^15^N compared to bulk biomass. The depletion of δ^15^N of lipid extracts compared to bulk biomass seems to be higher with organisms of a higher trophic level, the reasons for which need to be further explored.

## Supporting Information

S1 Table Stable nitrogen isotope values (δ^15^N), C:N ratios, and lipid content of bulk and residual biomass, and total lipid extracts of samples used in this study. Samples highlighted in yellow indicate those used for amino acid analysis.
(XLSX)

S2 Table Fatty acid composition, indicated by number of carbons and degree of unsaturation, of most abundant intact polar lipids (IPLs) in different aquatic species in this study. n.d. = not detected. PC: Phosphatidylcholine; PE: Phosphatidylethanolamine; MMPE: monomethyl–PE; PI: Phosphatidylinositol; Plasmalogen: Fatty acid with vinyl linkage to glycerol backbone; Lyso: IPL where one fatty acid has been removed (lysed); Sphingo: Sphingobase. See Fig 3 for structures.
(DOCX)

## References

[1] Boecklen WJ, Yarnes CT, Cook BA, James AC. On the use of stable isotopes in trophic ecology. Annu Rev Ecol Evol Syst. 2011;42: 411–440. 10.1146/annurev-ecolsys-102209-144726

[2] Middelburg JJ. Stable isotopes dissect aquatic food webs from the top to the bottom. Biogeosciences. 2014;11: 2357–2371. 10.5194/bg-11-2357-2014

[3] Fry B, Scalan RS, Parker PL. Stable carbon isotope evidence for two sources of organic matter in coastal sediments: seagrasses and plankton. Geochim Cosmochim Acta. 1977;41: 1875–1877. 10.1016/0016-7037(77)90218-6

[4] Hobson KA. Tracing origins and migration of wildlife using stable isotopes: a review. Oecologia. 1999;120: 314–326. 28308009 10.1007/s004420050865

[5] DeNiro MJ, Epstein S. Influence of diet on the distribution of nitrogen isotopes in animals. Geochim Cosmochim Acta. 1981;45: 341–351. 10.1016/0016-7037(81)90244-1

[6] Minagawa M, Wada E. Stepwise enrichment of ^15^N along food chains: Further evidence and the relation between δ^15^N and animal age. Geochim Cosmochim Acta. 1984;48: 1135–1140. 10.1016/0016-7037(84)90204-7

[7] McClelland J, Montoya J. Trophic relationships and the nitrogen isotopic composition of amino acids in plankton. Ecology. 2002;83: 2173–2180.

[8] Chikaraishi Y, Kashiyama Y, Ogawa NO, Kitazato H, Ohkouchi N. Metabolic control of nitrogen isotope composition of amino acids in macroalgae and gastropods: implications for aquatic food web studies. Mar Ecol Prog Ser. 2007;342: 85–90. 10.3354/meps342085

[9] McCarthy MD, Benner R, Lee C, Fogel ML. Amino acid nitrogen isotopic fractionation patterns as indicators of heterotrophy in plankton, particulate, and dissolved organic matter. Geochim Cosmochim Acta. 2007;71: 4727–4744. 10.1016/j.gca.2007.06.061

[10] DeNiro MJ, Epstein S. Mechanism of carbon isotope fractionation associated with lipid synthesis. Science. 1977;197: 261–263. 327543 10.1126/science.327543

[11] Monson KD, Hayes JM. Carbon isotopic fractionation in the biosynthesis of bacterial fatty acids. Ozonolysis of unsaturated fatty acids as a means of determining the intramolecular distribution of carbon isotopes. Geochim Cosmochim Acta. 1982;46: 139–149. 10.1016/0016-7037(82)90241-1

[12] Pinnegar JK, Polunin NVC. Differential fractionation of δ13C and δ15N among fish tissues: implications for the study of trophic interactions. Funct Ecol. 1999;13: 225–231. 10.1046/j.1365-2435.1999.00301.x

[13] Sotiropoulos MA, Tonn WM, Wassenaar LI, Sotiropoulos MA, Tonn WM, Wassenaar LI. Effects of lipid extraction on stable carbon and nitrogen isotope analyses of fish tissues: potential consequences for food web studies, Effects of lipid extraction on stable carbon and nitrogen isotope analyses of fish tissues: potential consequences for food web studies. Ecol Freshw Fish. 2004;13: 155–160. 10.1111/j.1600-0633.2004.00056.x

[14] Murry BA, Farrell JM, Teece MA, Smyntek PM. Effect of lipid extraction on the interpretation of fish community trophic relationships determined by stable carbon and nitrogen isotopes. Can J Fish Aquat Sci. 2006;63: 2167–2172. 10.1139/f06-116

[15] Elsdon TS, Ayvazian S, McMahon KW, Thorrold SR. Experimental evaluation of stable isotope fractionation in fish muscle and otoliths. Mar Ecol Prog Ser. 2010;408: 195–205. 10.3354/meps08518

[16] McConnaughey T, McRoy CP. Food-web structure and the fractionation of carbon isotopes in the Bering Sea. Mar Biol. 1979;53: 257–262. 10.1007/BF00952434

[17] Logan JM, Jardine TD, Miller TJ, Bunn SE, Cunjak RA, Lutcavage ME. Lipid corrections in carbon and nitrogen stable isotope analyses: comparison of chemical extraction and modelling methods. J Anim Ecol. 2008;77: 838–846. 10.1111/j.1365-2656.2008.01394.x 18489570

[18] Svensson E, Freitas V, Schouten S, Middelburg JJ, van der Veer HW, Sinninghe Damsté JS. Comparison of the stable carbon and nitrogen isotopic values of gill and white muscle tissue of fish. J Exp Mar Biol Ecol. 2014;457: 173–179. 10.1016/j.jembe.2014.04.014

[19] Vander Zanden MJ, Rasmussen JB. Variation in δ^15^N and δ^13^C trophic fractionation: implications for aquatic food web studies. Limnol Oceanogr. 2001; 2061–2066.

[20] Herzka SZ, Holt GJ. Changes in isotopic composition of red drum (*Sciaenops ocellatus*) larvae in response to dietary shifts: potential applications to settlement studies. Can J Fish Aquat Sci. 2000;57: 137–147. 10.1139/f99-174

[21] Logan JM, Lutcavage ME. A comparison of carbon and nitrogen stable isotope ratios of fish tissues following lipid extractions with non-polar and traditional chloroform/methanol solvent systems. Rapid Commun Mass Spectrom. 2008;22: 1081–1086. 10.1002/rcm.3471 18327856

[22] van der Veer H, Koot J, Aarts G, Dekker R, Diderich W, Freitas V, et al Long-term trends in juvenile flatfish indicate a dramatic reduction in nursery function of the Balgzand intertidal, Dutch Wadden Sea. Mar Ecol Prog Ser. 2011;434: 143–154. 10.3354/meps09209

[23] Sturt HF, Summons RE, Smith K, Elvert M, Hinrichs K-U. Intact polar membrane lipids in prokaryotes and sediments deciphered by high-performance liquid chromatography/electrospray ionization multistage mass spectrometry—new biomarkers for biogeochemistry and microbial ecology. Rapid Commun Mass Spectrom. 2004;18: 617–628. 10.1002/rcm.1378 15052572

[24] Schouten S, Hopmans EC, Baas M, Boumann H, Standfest S, Konneke M, et al Intact membrane lipids of “*Candidatus Nitrosopumilus maritimus*”, a cultivated representative of the cosmopolitan mesophilic Group I Crenarchaeota. Appl Environ Microbiol. 2008;74: 2433–2440. 10.1128/AEM.01709-07 18296531 PMC2293165

[25] Bale NJ, Villanueva L, Hopmans EC, Schouten S, Sinninghe Damsté JS. Different seasonality of pelagic and benthic Thaumarchaeota in the North Sea. Biogeosciences. 2013;10: 7195–7206. 10.5194/bg-10-7195-2013

[26] Brandsma J, Hopmans EC, Brussaard CPD, Witte HJ, Schouten S, Sinninghe Damsté JS. Spatial distribution of intact polar lipids in North Sea surface waters: Relationship with environmental conditions and microbial community composition. Limnol Oceanogr. 2012;57: 959–973. 10.4319/lo.2012.57.4.0959

[27] Schimmelmann A, Albertino A, Sauer PE, Qi H, Molinie R, Mesnard F. Nicotine, acetanilide and urea multi-level ^2^H-, ^13^C- and ^15^N-abundance reference materials for continuous-flow isotope ratio mass spectrometry. Rapid Commun Mass Spectrom. 2009;23: 3513–3521. 10.1002/rcm.4277 19844968

[28] Metges CC, Petzke K, Hennig U. Gas chromatography/combustion/isotope ratio mass spectrometric comparison of N -acetyl- and N-pivaloyl amino acid esters to measure ^15^N isotopic abundances in physiological samples: a pilot study on amino acid synthesis in the upper gastro-intestinal tract of minipigs. J Mass Spectrom. 1996;31: 367–376. 10.1002/(SICI)1096-9888(199604)31:4<367::AID-JMS310>3.0.CO;2-V 8799283

[29] Chikaraishi Y, Ogawa NO, Kashiyama Y, Takano Y, Suga H, Tomitani A, et al Determination of aquatic food-web structure based on compound-specific nitrogen isotopic composition of amino acids. Limnol Oceanogr Methods. 2009;7: 740–750. 10.4319/lom.2009.7.740

[30] Bodin N, Le Loc’h F, Hily C. Effect of lipid removal on carbon and nitrogen stable isotope ratios in crustacean tissues. J Exp Mar Biol Ecol. 2007;341: 168–175. 10.1016/j.jembe.2006.09.008

[31] Ingram T, Matthews B, Harrod C, Stephens T, Grey J, Markel R, et al Lipid extraction has little effect on the δ^15^N of aquatic consumers. Limnol Oceanogr Methods. 2007;5: 338–343.

[32] Cole LK, Vance JE, Vance DE. Phosphatidylcholine biosynthesis and lipoprotein metabolism. Biochim Biophys Acta BBA—Mol Cell Biol Lipids. 2012;1821: 754–761. 10.1016/j.bbalip.2011.09.009 21979151

[33] Elwyn D, Weissbach A, Henry SS, Sprinson DB. The biosynthesis of choline from serine and related compounds. J Biol Chem. 1955;213: 281–295. 14353926

[34] Stekol JA. Biosynthesis of Choline and Betaine. Am J Clin Nutr. 1958;6: 200–215. 13533306 10.1093/ajcn/6.3.200

[35] Germain LR, Koch PL, Harvey J, McCarthy MD. Nitrogen isotope fractionation in amino acids from harbor seals: implications for compound-specific trophic position calculations. Mar Ecol Prog Ser. 2013;482: 265–277. 10.3354/meps10257

[36] Chikaraishi Y, Steffan SA, Ogawa NO, Ishikawa NF, Sasaki Y, Tsuchiya M, et al High-resolution food webs based on nitrogen isotopic composition of amino acids. Ecol Evol. 2014;4: 2423–2449. 10.1002/ece3.1103 25360278 PMC4203290

[37] Hoen DK, Kim SL, Hussey NE, Wallsgrove NJ, Drazen JC, Popp BN. Amino acid ^15^N trophic enrichment factors of four large carnivorous fishes. J Exp Mar Biol Ecol. 2014;453: 76–83. 10.1016/j.jembe.2014.01.006

