# Supplementary material for: Factors Controlling the Stable Nitrogen Isotopic Composition (δ15N) of Lipids in Marine Animals
Source: PLoS One. 2016 Jan 5;11(1):e0146321. doi: 10.1371/journal.pone.0146321 (PMC4701503; doi:10.1371/journal.pone.0146321)
Supplement: S2 Table — n.d. = not detected. PC: Phosphatidylcholine; PE: Phosphatidylethanolamine; MMPE: monomethyl–PE; PI: Phosphatidylinositol; Plasmalogen: Fatty acid with vinyl linkage to glycerol backbone; Lyso: IPL where one fatty acid has been removed (lysed); Sphingo: Sphingobase. See Fig 3 for structures. (DOCX) [file pone.0146321.s002.docx]

Table S2. Fatty acid composition, indicated by number of carbons and degree of unsaturation, of most abundant intact polar lipids (IPLs) in different aquatic species in this study. n.d. = not detected. PC: Phosphatidycholine; PE: Phosphatidylethanolamine; MMPE: monomethyl–PE; PI: Phosphatidylinositol; Plasmalogen: Fatty acid with vinyl linkage to glycerol backbone; Lyso: IPL where one fatty acid has been removed (lysed); Sphingo: Sphingobase. See Figure 3 for structures.

|  | **Atlantic herring** | | **Brown trout** | | **Green shore crab** | **Common cockle** | **Pacific oyster** | **Lugworm** |
| --- | --- | --- | --- | --- | --- | --- | --- | --- |
|  | **muscle** | **gill** | **muscle** | **gill** | **muscle** | **head** | **muscle** | **muscle** |
| **PC** | 38:6  34:1 | 34:1  32:1  40:2 | 38:6  36:6  34:1 | 34:1  32:1  36:1  36:2 | 34:1  32:1 | 36:2  34:1  32:0  32:1  34:2 | 38:4-38:6  36:6  32:1  37:5 | 34:1  36:5  32:0  32:1  30:0 |
| **Plasmalogen–PC** | n.d. | 34:0 | 38:5 | 34:0  36:0 | 36:3  32:0  38:4  40:5 | n.d. | n.d. | n.d. |
| **Sphingo–PC** | n.d. | n.d. | 42:2 | 42:2  34:1  32:1 | 32:1  34:1  36:1 | n.d. | n.d. | n.d. |
| **PE** | n.d. | n.d. | n.d. | n.d. | 38:5  38:6 | n.d. | 38:3  38:4  40:5 | 40:3 |
| **MMPE** | n.d. | n.d. | n.d. | n.d. | n.d. | n.d. | 38:3 |  |
| **Betaine** | n.d. | n.d. | n.d. | n.d. | n.d. | n.d. | 32:1 | n.d. |
| **PI** | n.d. | n.d. | 41:4 | n.d. | n.d. | 36:6 | n.d. | n.d. |
